# Prevalence of hybrid TLR4^+^M2 monocytes/macrophages in peripheral blood and lung of systemic sclerosis patients with interstitial lung disease

**DOI:** 10.3389/fimmu.2024.1488867

**Published:** 2024-11-20

**Authors:** Emanuele Gotelli, Stefano Soldano, Carol Feghali-Bostwick, Paola Montagna, Rosanna Campitiello, Paola Contini, Marco Mora, Roberto Benelli, Elvis Hysa, Sabrina Paolino, Carmen Pizzorni, Alberto Sulli, Vanessa Smith, Maurizio Cutolo

**Affiliations:** ^1^ Laboratory of Experimental Rheumatology and Academic Division of Clinical Rheumatology, Department of Internal Medicine and Medical Specialties (Di.M.I.), University of Genova, Genova, Italy; ^2^ Department of Medicine, Division of Rheumatology and Immunology, Medical University of South Carolina, Charleston, SC, United States; ^3^ IRCCS Ospedale Policlinico San Martino, Genova, Italy; ^4^ Unit of Clinical Immunology and Translational Medicine, Department of Internal Medicine and Medical Specialties (Di.M.I.), University of Genova, Genova, Italy; ^5^ U.O. Anatomia Patologica Ospedaliera, IRCCS Ospedale Policlinico San Martino, Genova, Italy; ^6^ SSD Oncologia Molecolare e Angiogenesi, IRCCS Ospedale Policlinico San Martino, Genova, Italy; ^7^ Department of Experimental Medicine (DIMES), University of Genova, Genova, Italy; ^8^ Department of Rheumatology, Ghent University Hospital, University of Ghent, Ghent, Belgium; ^9^ Department of Internal Medicine, Ghent University Hospital, University of Ghent, Ghent, Belgium; ^10^ Unit for Molecular Immunology and Inflammation, Flemish Institute for Biotechnology, Inflammation Research Center, Ghent, Belgium

**Keywords:** systemic sclerosis, interstitial lung disease, connective tissue diseases, monocytes, macrophages, fibrosis

## Abstract

**Introduction:**

Systemic sclerosis (SSc) is a complex autoimmune connective tissue disease characterized by microvascular damage, immune system reactivity and progressive fibrosis of skin and internal organs. Interstitial lung disease is the leading cause of death for SSc patients (SSc-ILD), and the process of lung fibrosis involves also circulating monocytes and alveolar macrophages.

**Methods:**

Current study aimed to identify monocyte/macrophage phenotypes in lung and peripheral blood of SSc-ILD patients by immunostaining and flow cytometry, respectively. Single immunostaining was performed using primary antibodies against CD68 (pan-macrophage marker), CD80, CD86, TLR4 (M1 markers), CD163, CD204, and CD206 (M2 markers). Flow cytometry analysis included the evaluation of CD45, CD14, CD16 (monocyte lineage), CD1c (dendritic lineage), together with M1 and M2 activation markers on circulating monocytes. Protein synthesis of TLR4 and M2 markers was also investigated in cultured monocytes-derived macrophages (MDMs) from SSc-ILD patients by Western Blotting.

**Results:**

Lung samples were obtained from 9 SSc-ILD patients (50 ± 9 years old) and 5 control non-SSc patients without lung fibrosis (58 ± 23 years old). Alveolar macrophages (CD68^+^ cells) showed a significantly higher positivity of M1 and M2 markers in SSc-ILD lung samples than in controls (p<0.05 for CD80, p<0.01 for CD86, p<0.001 for CD68, p<0.0001 for TLR4, CD163, CD204 and CD206). In CD68 positive areas of SSc-ILD samples, a significantly higher percentage of TLR4, CD163, CD204, and CD206 positive cells was observed compared to CD80 and CD86 positive cells (p<0.001 in both cases), suggesting the possible presence of hybrid TLR4^+^M2 macrophages (CD68^+^CD80^-^CD86^-^TLR4^+^CD163^+^CD204^+^CD206^+^cells) in SSc-ILD samples. A second cohort of 26 SSc-ILD patients (63 ± 14 years old) and 14 SSc patients without ILD (63 ± 19 years old) was recruited for flow cytometry analysis of circulating monocytes. Again, a significantly higher percentage of hybrid TLR4^+^M2 monocytes (CD1c^-^CD80^-^TLR4^+^CD163^+^CD204^+^CD206^+^cells) was found in SSc-ILD positive than SSc-ILD negative patients (p<0.05). Moreover, the protein synthesis of TLR4 and M2 markers was also found higher in cultured MDMs obtained from SSc-ILD patients than in MDMs from SSc patients without ILD and this increase was significantly higher for CD163 (p<0.05) and CD206 (p<0.01).

**Conclusions:**

The presence of hybrid TLR4^+^M2 markers on both circulating monocytes and resident lung macrophages in SSc-ILD patients, is reported for the first time. Therefore, the detection of circulating hybrid TLR4^+^M2 monocytes in SSc-ILD might represent a further potential biomarker of progressive organ fibrosis, to be searched in blood samples of SSc patients.

## 1 Introduction

Systemic sclerosis (SSc) is a complex autoimmune connective tissue disease characterized by microvascular damage, immune system reactivity and progressive fibrosis of skin and internal organs (1, 2). Interstitial lung abnormalities are detected by high resolution computed tomography (HRCT) in up to 80% of SSc-patients and half of these patients develop clinically significant SSc-interstitial lung disease (ILD) (3).

The pathogenesis of SSc-ILD is still partially unknown (4). However, microvascular damage of the alveolo-capillary membrane with endothelial-to-mesenchymal transition is pivotal in this process (5, 6). It causes the release of pro-inflammatory cytokines, such as interferon gamma (IFN-γ) and interleukin (IL)-6 by innate immune cells and the early activation of classically pro-inflammatory macrophages (M1 macrophages) with consequent alveolar damage (5, 6).

These cytokines activate T helper 2 (T_h_2) lymphocytes that produce IL-4, IL-10, IL-13, C-C motif chemokine ligand-18 (CCL-18) and stimulate pro-fibrotic alternatively activated macrophages (M2 macrophages) with the release of profibrotic transforming growth factor β (TGF-β), finally promoting the activation of fibroblasts and their differentiation into myofibroblasts (7, 8). Myofibroblasts produce a large amount of extracellular matrix components (ECM), primarily type I collagen, fibronectin, vimentin and fibroblast growth factors (FGFs) that further promote M2 polarization, perpetuating lung fibrosis (9).

Monocytes, macrophages and their different functional polarization are actively involved in the pathogenesis of a large plethora of autoimmune rheumatic diseases (i.e., rheumatoid arthritis, psoriatic arthritis, Sjögren’s syndrome, systemic lupus erythematosus), including SSc (10–13).

Circulating monocytes of SSc-ILD patients show a prevalent M2 phenotype, expressing CD163, CD204 (macrophage scavenger receptors and M2 markers) and CD206 (mannose receptor-1) as M2 cell membrane markers, and producing IL-10 and CCL-18 (14–17). Moreover, SSc circulating monocytes secrete significantly more soluble fibronectin than those from healthy subjects (HSs) (18).

Of note, a subpopulation of circulating monocytes co-expressing both M1 and M2 markers has been already described in SSc-ILD patients by our group (19). These “hybrid” monocytes (CD14^+^CD80^+^CD86^+^TLR4^+^CD163^+^CD204^+^CD206^+^cells) are significantly more expressed in the peripheral blood of SSc-patients than HSs and are associated with clinically overt SSc-ILD (20). On the other hand, monocyte-derived macrophages (CD163^+^cells, CD206^+^cells) from SSc-ILD patients show M2 polarization and release TGF-β, IL-6, CCL-18 and osteopontin, that promote epithelial-to-mesenchymal transition (EMT), fibroblast-to-myofibroblast differentiation, and the progressive lung fibrotic process (21–25).

To date, the presence of “hybrid” macrophages in the lung tissue of SSc-ILD patients has never been investigated. So, the current study analyzes the macrophage phenotype distribution (M1, “hybrid” M1/M2 and M2) in SSc-ILD samples, compared with controls, by immunostaining, together with the monocyte phenotype distribution (M1, “hybrid” M1/M2 and M2) in the peripheral blood of a second cohort of SSc-patients (with or without SSc-ILD) by flow cytometry.

## 2 Materials and methods

### 2.1 Sampling from SSc-patients and controls

SSc-ILD samples were collected from SSc-patients undergoing lung transplantation for ILD at the University of Pittsburgh Medical Center, Pittsburgh, Pennsylvania (USA) under a protocol approved by the Institutional Review Board of the University (#970946). Control lung samples were obtained from non-SSc patients without lung fibrosing conditions that underwent lung biopsy for diagnostic purposes at the IRCCS Ospedale Policlinico San Martino, Genova, Italy.

Due to the technical limits to get and then analyze in short term fresh blood samples at the site of the surgical center, whole blood samples to perform the flow cytometry analysis were obtained from 40 SSc patients (26 with SSc-ILD and 14 without SSc-ILD) and 15 voluntary HSs at the Division of Clinical Rheumatology, IRCCS Ospedale Policlinico San Martino, Genova, Italy.

All SSc patients met 2013 ACR/EULAR classification criteria for SSc (26) and were treated with standard therapies, mainly immunosuppressors, according to their disease condition and severity (**Tables 1**, **2**). Instrumental examinations were part of their regular follow-up of the disease and nailfold videocapillaroscopy (NVC) was analyzed and quantified following international validated methods (27, 28).

**Table 1:** Demographic data of SSc-ILD patients with pulmonary function tests at time of lung transplantation and non-SSc patients.

	SSc-ILD patients (n=9)	Non-SSc controls (n=5)
Age (years, mean ± SD)	50 ± 9	58 ± 23
Gender (Female/Male)	6/3	2/3
Ethnicity (Caucasian/Afro-American)	7/2	5/0
Smokers/Non-smokers	2/7	1/4
Pneumothorax/limited lung cancer	0/0	2/3
PULMONARY FUNCTION TESTS		
FVC (reference percentage, mean value ± SD)	39.6 ± 12.3	–
FEV1 (reference percentage, mean value ± SD)	45.5 ± 14.1	–
TLC (reference percentage, mean value ± SD)	37.6 ± 9.0	–
DLCO (reference percentage, mean value ± SD)	17.4 ± 4.6	–
IMMUNOSUPPRESSIVE – ANTIFIBROTIC DRUGS		
Mycophenolate mofetil	4	–
Calcineurin inhibitors	4	–
Basiliximab	2	–
Alemtuzumab	2	–
Nintedanib	0	–

DLCO, Diffusion Lung Carbon Monoxide; FEV1, Forced Expiratory Volume in the first second; FVC, Forced Vital Capacity; ILD, Interstitial Lung Disease; SD, Standard Deviation; SSc, Systemic sclerosis; TLC, Total Lung Capacity.

**Table 2:** Demographic data and pulmonary function tests of SSc patients for flow cytometry monocytes analysis at time of blood sample.

	SSc-ILD patients (n=26)	SSc patients without ILD (n=14)
Age (years, mean ± SD)	63 ± 14	63 ± 19
Gender (Female/Male)	22/4	14/0
Ethnicity (Caucasian/Afro-American)	26/0	13/1
Smokers/Non-smokers	1/25	1/13
AUTOANTIBODIES		
Anti-centromere positivity, %	4 (15%)	8 (57%)
Anti-topoisomerase I (Scl-70) positivity, %	17 (65%)	1 (7%)
Anti-RNA III polymerase positivity, %	0 (0%)	0 (0%)
Other, %	5 (20%)	5 (36%)
NAILFOLD VIDEOCAPILLAROSCOPY		
“Early” pattern, %	4 (15%)	1 (7%)
“Active” pattern, %	9 (35%)	12 (86%)
“Late” pattern, %	13 (50%)	1 (7%)
PULMONARY FUNCTION TESTS		
FVC (reference percentage, mean value ± SD)	84 ± 29	102 ± 21
DLCO/VA (reference percentage, mean value ± SD)	74 ± 23	79 ± 14
IMMUNOSUPPRESSIVE-ANTIFIBROTIC DRUGS		
Mycophenolate mofetil, %	12 (46%)	3 (21%)
Cyclophosphamide, %	1 (4%)	0
Rituximab, %	1 (4%)	0
Nintedanib, %	3 (12%)	0

DLCO, Diffusion Lung Carbon Monoxide; FVC, Forced Vital Capacity; ILD, Interstitial Lung Disease; SD, Standard Deviation; SSc, Systemic Sclerosis; VA, Alveolar Volume.

### 2.2 Histopathological assessment

Lung tissues were fixed in 10% formalin, embedded in paraffin, and then cryostat-sectioned to obtain serial sections (4 μm thick).

A section of each SSc-ILD patient and control was stained with Masson’s trichrome staining to identify collagen distribution, cells infiltrate, and to detect the degree of tissue fibrosis.

Other serial sections were incubated for single immunostainings with the following primary antibodies: mouse anti-human CD68 (pan specific macrophage marker), mouse anti-human CD80, CD86, TLR4 (M1 markers), rabbit anti-human CD163 and CD206, mouse anti-human CD204 (M2 markers). Primary antibodies were detected with ready-to-use conjugated secondary antibodies, using 3’3’-diaminobenzidine (DAB) as chromogenic substrate. Further information regarding primary antibodies used for single immunostaining are provided in **Supplementary Material 1**. Histology sections were digitalized using Leica AT2 scanner.

Fibrosis of lung samples was semi-automated quantified (0 = absence of fibrosis, 1 = complete fibrosis) using open-source version of Orbit Image Analysis software (29).

The percentage of cells positive for CD68, CD80, CD86, TLR4, CD163, CD204, CD206 in the alveoli of SSc-ILD lungs were quantified by the Image Scope 12.3 Software (30).

### 2.3 Flow cytometry and gating strategy

To define the peripheral monocyte lineage, surface markers CD14, CD16 and CD45 were investigated using conjugated primary antibodies anti-human CD14-FITC, CD16-APC AlexaFluor 700, and CD45-Krome Orange.

Surface markers CD80 and TLR4 were investigated using conjugated primary antibodies anti-human CD80-PEVio770 and TLR4-BV421 to characterize M1 phenotype from circulating monocytes.

Surface markers CD163, CD204 and CD206 were investigated using conjugated primary antibodies anti-human CD163-PEVio615, CD204-PE and CD206-PerCPVio700 to characterize M2 phenotype from circulating monocytes.

Surface marker CD1c was investigated using the conjugated primary anti-human CD1c-APC Cy7 antibody to exclude the presence of dendritic cells.

Further information regarding primary antibodies used for flow cytometry analysis are provided in **Supplementary Material 1**.

Flow cytometry analysis was performed using Navios Flow Cytometer and Kaluza analysis software (Beckman Coulter, Brea, California, USA), evaluating a total of 5x10^6^ cells and detecting more than 30 events in the smallest subset investigated (31, 32).

**Figure 1:**
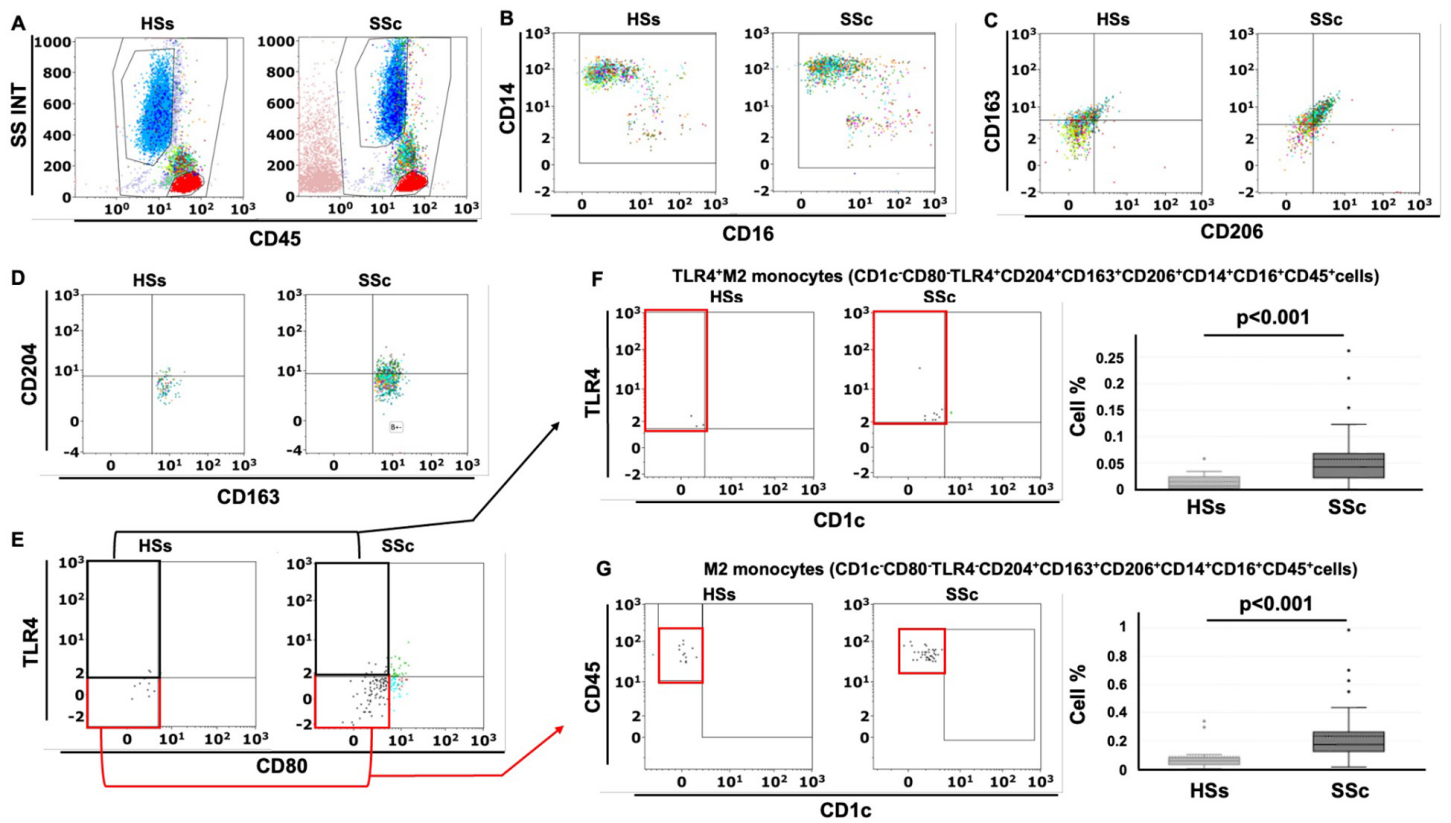
Gating strategy for the detection of circulating TLR4^+^M2 and M2 monocytes in systemic sclerosis patients (SSc pts) and healthy subjects (HSs) by flow cytometry. **(A)** Representative dot plot of leukocyte population using CD45 and physical parameters – Side scatter (SS) and Forward scatter (INT) – after removing cell debris **(B)** Flow cytometry scatter dot plot to identify circulating monocytes (CD45^+^CD14^+^CD16^+^cells) in the leukocyte population; **(C)** Flow cytometry scatter dot plot with quadrant regions of circulating CD163^+^CD206^+^ cells in the monocyte population; **(D)** CD204^+^CD163^+^CD206^+^cells in the monocyte population; **(E)** CD80^-^TLR4^-^CD204^+^CD163^+^CD206^+^cells (M2 monocytes) and CD80^-^TLR4^+^CD204^+^CD163^+^CD206^+^cells (TLR4^+^M2 monocytes) in the CD204^+^CD163^+^CD206^+^ cells; **(F, G)** Flow cytometry scatter dot plot and related box plot representation of the percentage of TLR4^+^M2 monocytes (CD1c^-^CD80^-^TLR4^+^CD163^+^CD204^+^CD206^+^cells) and M2 monocytes (CD1c^-^CD80^-^TLR4^-^CD163^+^CD204^+^CD206^+^cells) in HSs and SSc pts, excluding the presence of dendritic cells (CD1c^+^cells).

The gating strategy used in this study started from the detection of the monocyte population characterized as CD45^+^CD14^+^CD16^+^cells in the leukocyte population (CD45+cells) excluding both lymphocytes and granulocytes and is depicted in **Figure 1**. The value in the box plots of the percentage of the investigated subsets of circulating monocytes is referred to the leukocyte population (32).

### 2.4 Isolation and culture of circulating monocytes from SSc patients and HSs

Venous blood sample (20 mL) was collected from enrolled HSs (n=5), SSc patients without ILD (n=5) and SSc-ILD patients (n=11, not on treatment with nintedanib), and monocytes were isolated using the EasySep human monocyte enrichment kit without CD16 depletion (Stemcell Technologies, Vancouver, Canada), and their viability and purity were assessed by Flow Cytometry.

The isolated monocytes were plated in tissue culture dishes (Eppendorf, Hamburg, Germany) at the concentration of 2.5x10^6^ cells, stimulated with phorbol myristate acetate (PMA, 5 ng/mL; Sigma-Aldrich, St. Louis, Missouri, USA) and then maintained in growth medium (RPMI with 10% fetal bovine serum, 1% penicillin-streptomycin, and 1% L-glutamine, Euroclone, Milan, Italy) for 24 hours to induce their differentiation into MDMs. The cells were finally lysed to isolate proteins for the evaluation of the above-mentioned M2 markers and TLR4.

### 2.5 Western blotting

Proteins were isolated using the RNA/Protein Purification Plus kit (Norgen Biotech) and quantified by Bradford method. Subsequently, 10 μg of protein was separated via electrophoresis on pre-cast 4-20% gradient tris-glycine gels (GenScript, Piscataway, USA) and then transferred onto a nitrocellulose membrane (Bio-Rad, Milan, Italy). After 1 hour in blocking solution, membranes were incubated overnight at 4°C with primary antibodies against human CD204, TLR4 (dilution 1:400 and 1:200, respectively; Santa Cruz Biotechnologies, Dallas, Texas, USA), CD206, CD163 (dilution 1:500, Cell Signaling Technology, Danvers, Massachusetts, USA) and glyceraldehyde 3-phosphate dehydrogenase (GAPDH dilution 1:1.000; Santa Cruz Biotechnologies). Membranes were subsequently incubated with specific horseradish peroxidase-conjugated secondary antibodies (dilution 1:2.000; Cell Signaling Technology) for 1 hour at room temperature.

Protein synthesis was detected using an enhanced chemiluminescence system (SuperSignal West Pico PLUS Chemiluminescent Substrate, Thermo Scientific, Rockford, USA) and the densitometric analysis was performed by the UVITEC Image Analysis System (UVITEC, Cambridge, UK).

For each sample (SSc patients and HSs), the value of the protein synthesis of the investigated molecules was normalized to that of the GAPDH as housekeeping protein. The resulting value of each sample of SSc patients was then normalized to that of the corresponding HSs (considered as the unit value).

### 2.6 Statistical analysis

Statistical analysis was carried out using GraphPad Prism and Datatab^®^ Statistics Calculator. Continuous variables were reported as mean value and standard deviation (SD) or median, when appropriate, categorical variables as count and percentage. For comparing two group values, T-student and Levene test of variance equality have been performed while for groups that did not follow Gaussian distribution, the two-tailed Mann-Whitney U test was used. Spearman’s rank correlation was used to calculate the relationship between ordinal variables, whereas Pearson’s correlation analysis was used for metrically scaled variables. Any p values equal or lower than 0.05 were considered statistically significant.

### 2.7 Ethics statement

Lung sample analyses were carried out in accordance with the recommendations of the Medical University of South Carolina (Charleston, USA), University of Pittsburgh (Pennsylvania, USA) Institutional Review Boards (Protocol #970946) and IRCCS Ospedale Policlinico San Martino (Genova, Italy) with written informed consent from all patients as standard procedures.

The blood collection for flow cytometry investigation was approved by the Ethics Committee of IRCCS Ospedale Policlinico San Martino (273-reg-2015) and all patients signed the informed consent forms.

## 3 Results

### 3.1 SSc-ILD and control lung samples

SSc-ILD lung samples were obtained from nine SSc-ILD patients (ID samples: SSc-71, SSc-72, SSc-73, SSc-74, SSc-77, SSc-83, SSc-88, SSc-90, SSc-91; 6 females and 3 males, 50 ± 9 years old), while control lung samples were obtained from five non-SSc patients (ID samples: CNT-A3, CNT-A5, CNT-A6, CNT-D8, CNT-D9; 2 females, 3 males, 58 ± 23 years old) who underwent lung biopsy for pneumothorax (two cases) and early localized lung cancer (three cases). The lung biopsies of non-SSc patients were collected from the area not involved by the disease. Demographic data of SSc patients and controls are reported in **Table 1**.

### 3.2 Higher collagen deposition and cell infiltrates in stroma and alveoli of SSc-ILD lung than controls

Masson’s trichrome staining of SSc-ILD samples identified a massive collagen deposition and cell infiltrates in both stroma and alveoli of every sample, confirming the presence of lung fibrosis. Most alveoli were found collapsed and occluded by connective tissue and cellular infiltrates. On the contrary, Masson’s trichrome staining of control lungs showed low collagen deposition and cell infiltrates primarily in the stroma than in alveoli.

**Figure 2:**
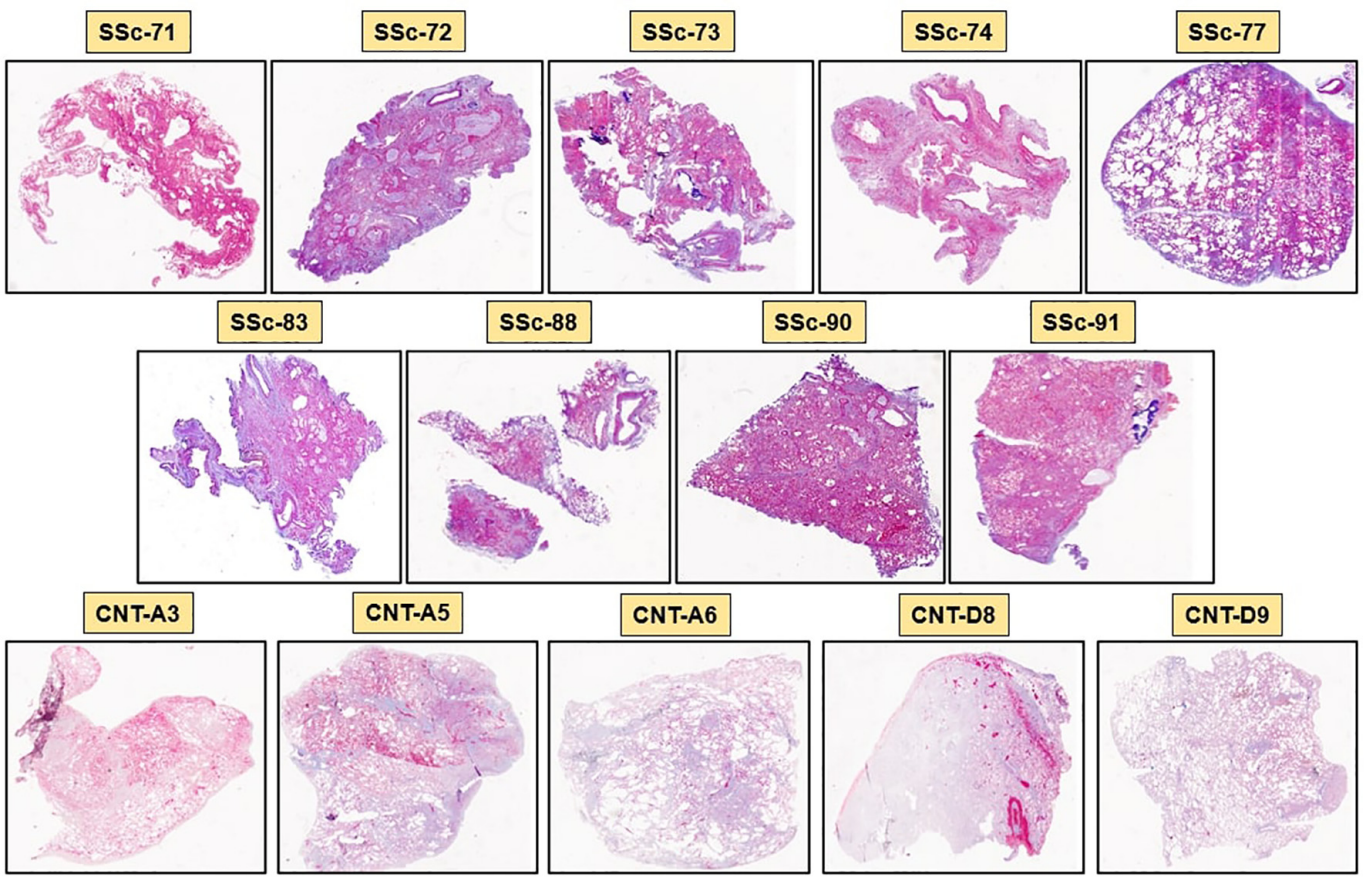
Masson’s trichrome staining of SSc-ILD lung samples (SSc-71, SSc-72, SSc-73, SSc-74, SSc-77, SSc-83, SSc-88, SSc-90, SSc-91) and control lung samples (CNT-A3, CNT-A5, CNT-A6, CNT-D8, CNT-D9). Blue color identifies collagen deposition, while violet color identifies cellular infiltrates in the stroma and in the alveoli (20x magnification).

The mean of software automatic quantification (0 = absence of fibrosis, 1 = complete fibrosis) of collagen tissue in SSc-ILD patients was 0.82 ± 0.12 compared to 0.27 ± 0.05 detected in controls (p<0.001). Masson’s trichrome staining of all SSc-ILD and control samples is depicted in **Figure 2**.

### 3.3 Higher percentage of cells expressing hybrid TLR4^+^M2 macrophage markers in SSc-ILD samples than controls

**Figure 3:**
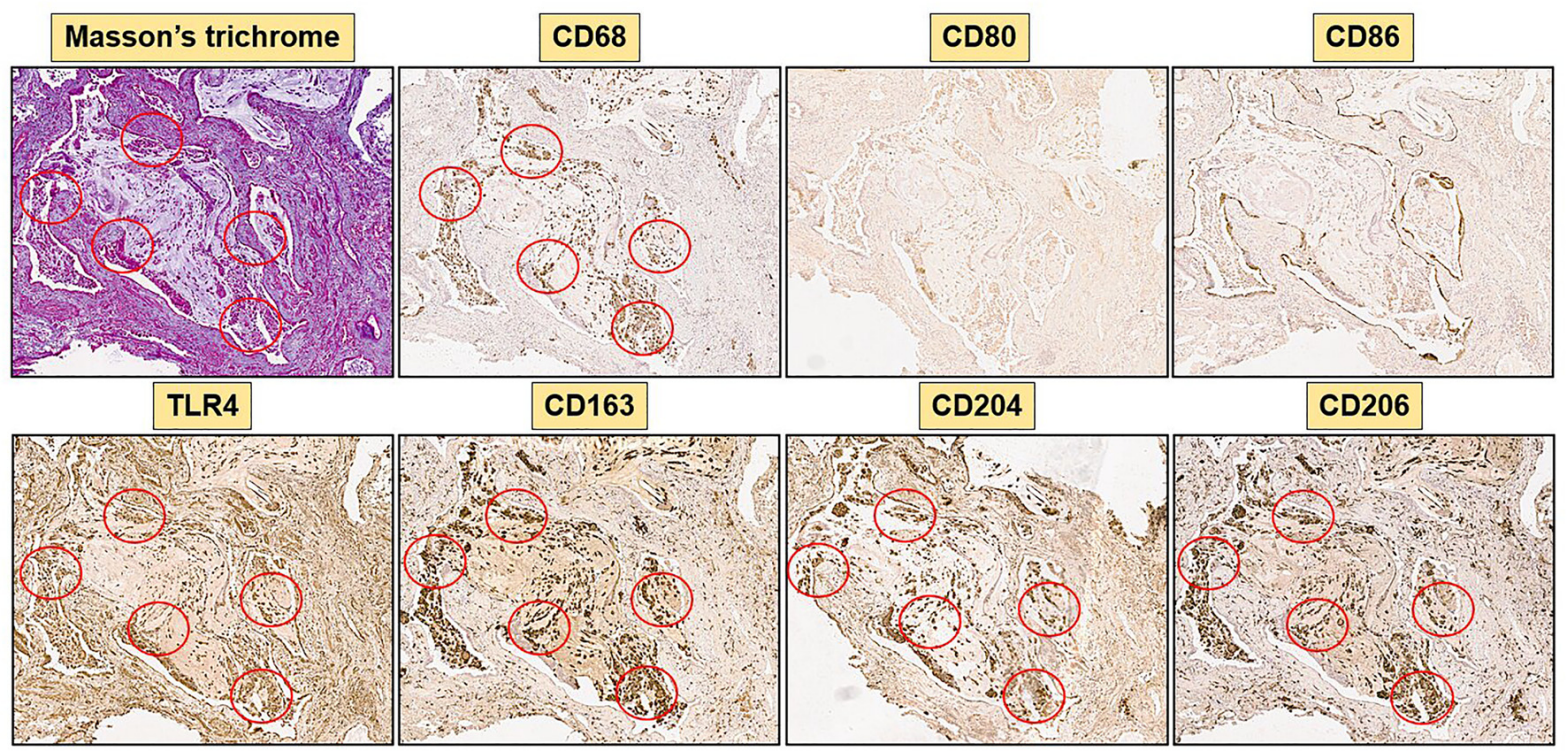
Immunohistochemistry of a SSc-ILD sample (SSc-83) with 40x magnification. Representative Masson’s trichrome and immunostaining for CD68, M1 markers (CD80, CD86, TLR4) and M2 markers (CD163, CD204, CD206) are depicted. Red circles highlight areas rich of collagen in Masson’s trichrome staining and areas of co-localization of TLR4 positive cells and M2 markers (CD163, CD204, CD206).

**Figure 4:**
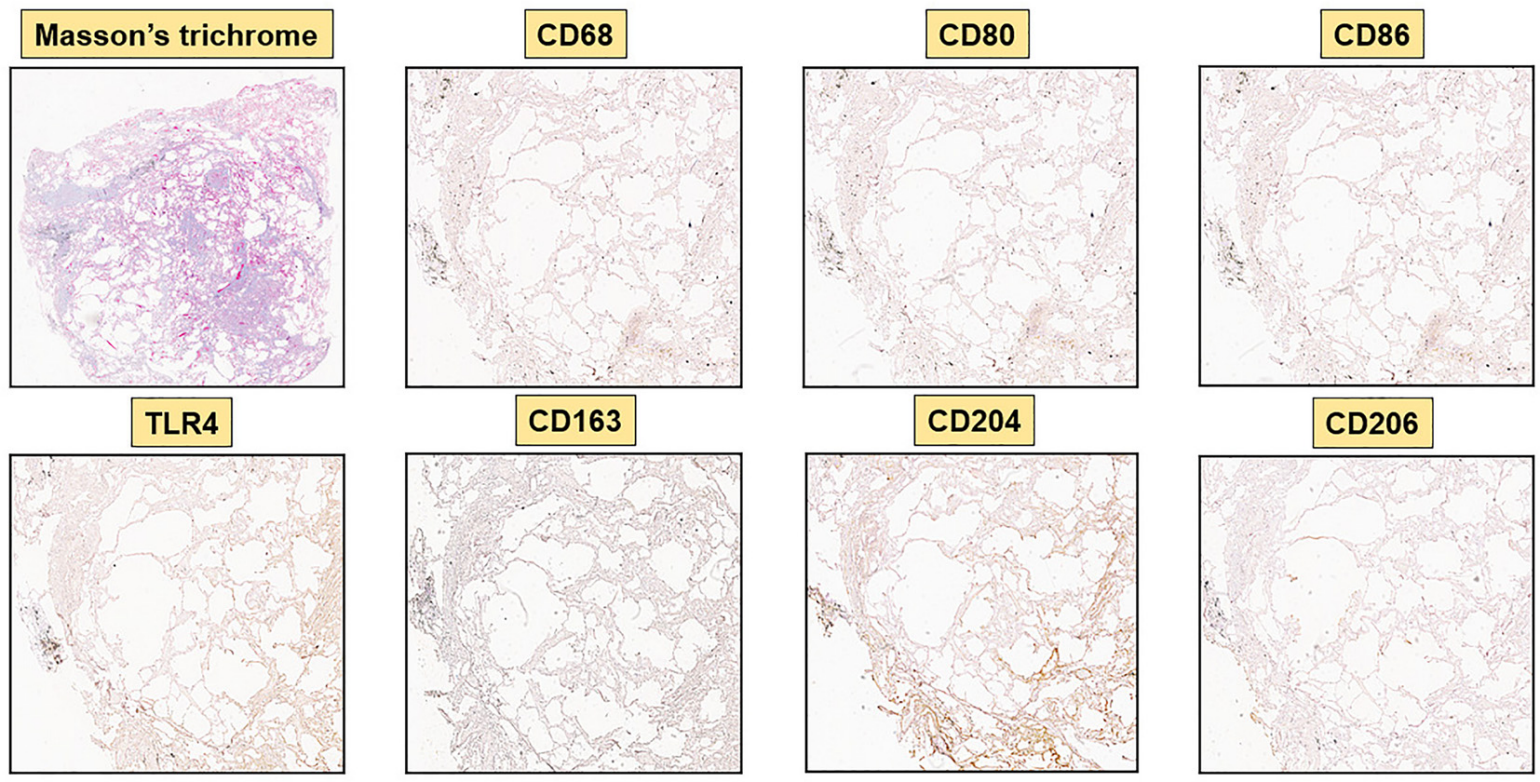
Immunohistochemistry of a control lung sample (CNT-A6) with 40x magnification. Representative Masson’s trichrome and immunostaining for CD68, M1 markers (CD80, CD86, TLR4) and M2 markers (CD163, CD204, CD206) are depicted. M1 and M2 markers are virtually absent.

**Figure 5:**
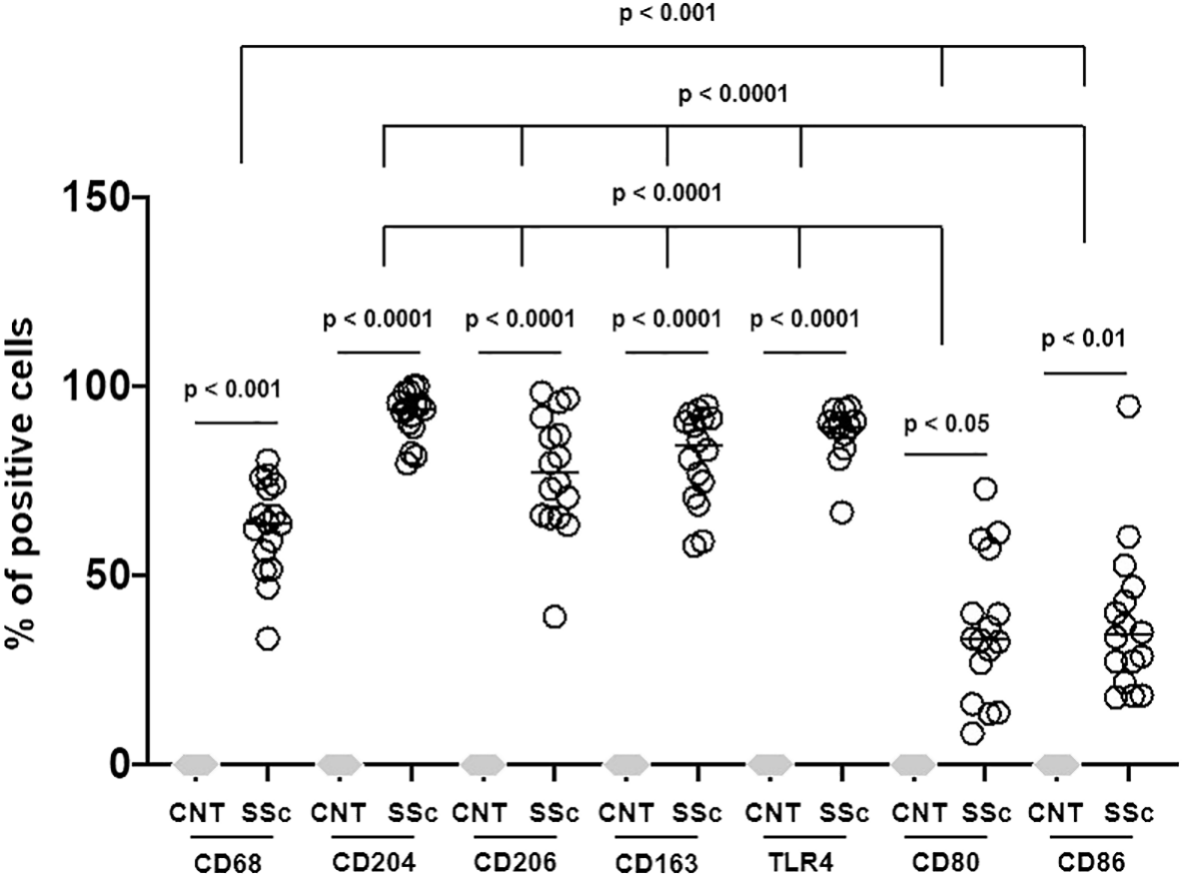
Percentage of positivity of all macrophage surface markers out of total cells in SSc-ILD lung samples versus controls (CNT). Three-quarters (almost 75%) of the cells found in the SSc-ILD lung samples belong to macrophage lineage. CD68, TLR4 (M1 marker), CD163 (M2 marker), CD204 (M2 marker) and CD206 (M2 marker) positive cells were found in the same areas. CD80 (M1 marker) and CD86 (M1 marker) positive cells were significantly less expressed than other markers investigated.

Immunostaining of SSc-ILD samples showed a significantly increased presence of CD68^+^cells in both stroma and alveoli compared to controls (p<0.001) (**Figures 3**–**5**).

Concerning the M2 phenotype markers, a relevant presence of CD204^+^cells, CD206^+^cells and CD163^+^cells was observed in the stroma and primarily in the alveoli of SSc-ILD patients: their percentages were significantly higher than those observed in controls (p<0.0001) (**Figures 3**–**5**). Of note, in the alveoli of SSc-ILD patients CD204^+^cells, CD206^+^cells and CD163^+^cells overlapped with CD68^+^cells; otherwise in the alveoli of controls these cells were absent (**Figures 3**–**5**).

Concerning the markers of M1 phenotype, in the lung of SSc-ILD patients, CD80^+^cells were observed in the stroma and alveoli, whereas CD86^+^cells were expressed in the alveolar wall layer facing the air and virtually absent in the stroma. The percentage of these cells was significantly higher compared to that of controls (p<0.05 for CD80^+^cells; p<0.01 for CD86^+^cells) (**Figures 3**–**5**). In SSc-ILD lungs, CD80^+^cells and CD86^+^cells overlapped with CD68^+^cells, even if they were significantly less frequent (p<0.001) (**Figures 3**–**5**).

TLR4^+^cells were found to be present in both stroma and alveoli of SSc-ILD patients, significantly more frequent than controls (p<0.0001) and overlapping with CD68^+^cells (**Figures 3**–**5**).

Of note, focusing on SSc-ILD alveoli, which were the compartment characterized by a higher presence of macrophage infiltrate (CD68^+^cells), there was a significant overlap between CD68^+^, CD204^+^, CD163^+^, CD206^+^ and TLR4^+^cells, revealing the likely the presence of TLR4^+^M2 macrophages. Conversely, CD80^+^ and CD86^+^cells were significantly less represented in these areas (**Figures 3**, **5**).

Hybrid TLR4^+^M2 macrophages, as well as CD80^+^ and CD86^+^cells, were absent in control lung samples (**Figures 4**, **5**).

### 3.4 Higher percentage of circulating hybrid TLR4^+^M2 and M2 monocytes in SSc-ILD positive than SSc-ILD negative patients

Following the results obtained from the lung samples analysis, 26 SSc patients with ILD (mean age 63 ± 14 years old), 14 SSc patients without ILD (mean age 63 ± 19 years old) and 15 age-matched HSs were enrolled for flow cytometry analysis to investigate the phenotype (hybrid TLR4^+^M2 and M2) of circulating monocytes. Demographic data of all SSc patients are resumed in **Table 2**. The flow cytometry analysis showed that the percentage of circulating hybrid TLR4^+^M2 monocytes (CD1c^-^CD80^-^TLR4^+^CD163^+^CD204^+^CD206^+^cells) as well as M2 monocytes (CD1c^-^CD80^-^TLR4^-^CD163^+^CD204^+^CD206^+^cells) in the leukocyte population were observed significantly increased in SSc patients compared to HSs (p<0.001) (**Figure 1**).

**Figure 6:**
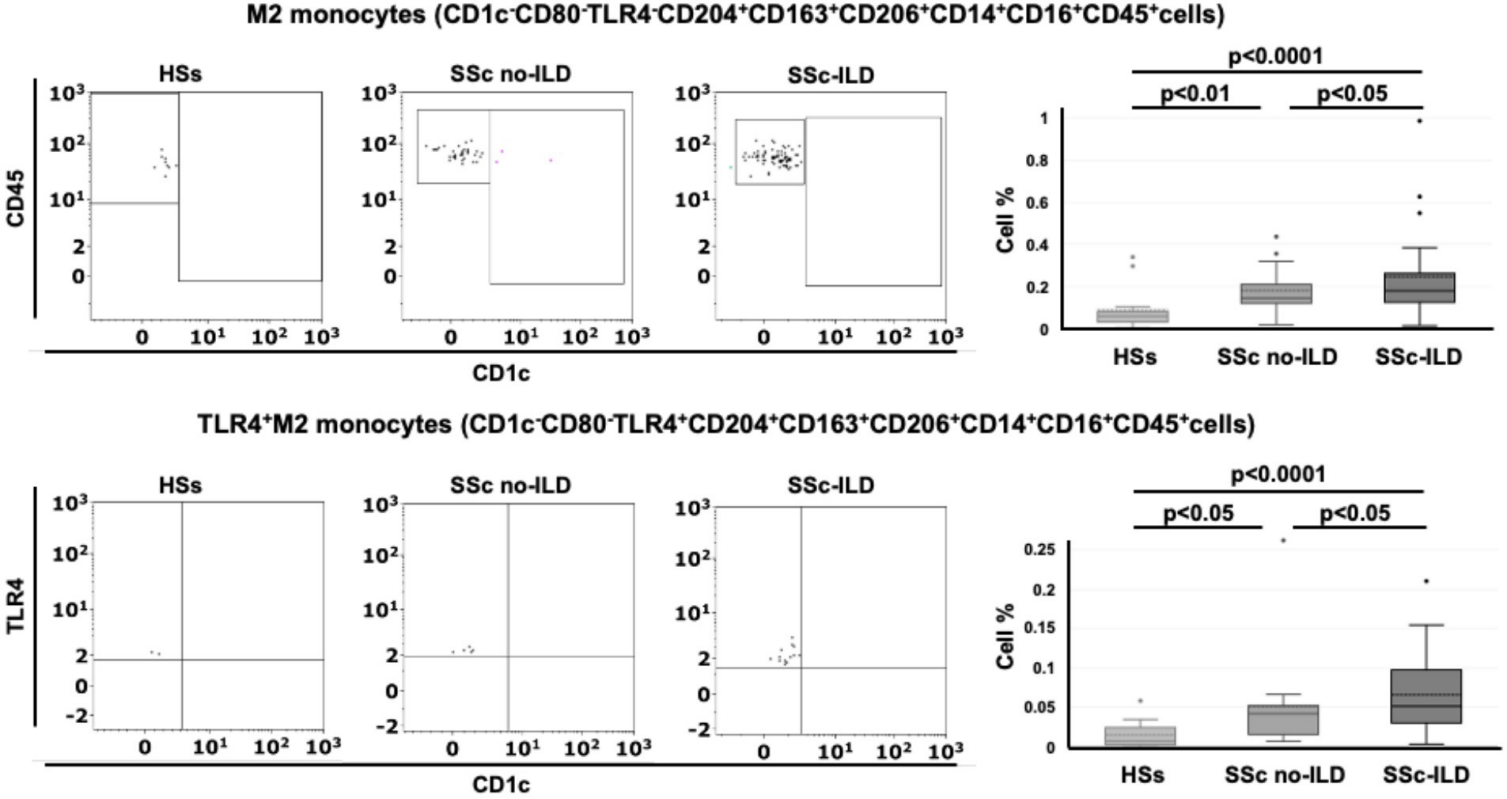
Flow cytometry analysis of circulating monocytes in systemic sclerosis (SSc) patients. Representative flow cytometry analysis to identify M2 monocytes (CD1c^-^CD80^-^TLR4^-^CD163^+^CD204^+^CD206^+^cells) and TLR4^+^M2 monocytes (CD1c^-^CD80^-^TLR4^+^CD163^+^CD204^+^CD206^+^cells) in the peripheral blood of healthy subjects (HSs) and SSc patients with ILD (SSc-ILD) or without interstitial lung disease (SSc no-ILD). The related box plot representation of the percentage of these cells was included.

The percentage of circulating hybrid TLR4^+^M2 and M2 monocytes was found significantly higher in SSc-ILD patients compared with SSc patients without ILD (p<0.05 for both cell populations) and HSs (p<0.0001 for both cell populations) (**Table 3**, **Figure 6**).

**Table 3:** Significant differences observed in the distribution of percentages of circulating cells expressing the monocyte/macrophage markers between systemic sclerosis (SSc) patients with or without interstitial lung disease (ILD).

Peripheral Cell phenotype	SSc-ILD+ (n=26)	SSc-ILD- (n=14)	P values
TLR4^+^M2 monocytes (CD1c^-^CD80^-^TLR4^+^CD163^+^CD204^+^CD206^+^CD14^+^CD16^+^CD45^+^ cells)	0.067 ± 0.049	0.034 ± 0.018	< 0.05
M2 monocytes (CD1c^-^CD80^-^TLR4^-^CD163^+^CD204^+^CD206^+^CD14^+^CD16^+^CD45^+^cells)	0.29 ± 0.22	0.16 ± 0.097	< 0.05

Values are expressed as means ± standard deviations of cells percentages. SSc-ILD+, systemic sclerosis patients with interstitial lung disease; SSc-ILD-, systemic sclerosis patients without interstitial lung disease.

Finally, a very small cohort of SSc-ILD positive patients (n=3) was on treatment with nintedanib. These patients were characterized by a median percentage of circulating M2 monocytes and hybrid TLR4^+^M2 monocytes lower than that observed in all others SSc-ILD patients (0.098 ± 0.05 *vs* 0.21 ± 0.2 and 0.016 ± 0.02 *vs* 0.05 ± 0.05, respectively).

### 3.5 Higher protein synthesis of TLR4 and M2 markers in cultured MDMs of SSc-ILD patients compared with SSc patients without ILD

Based on the results obtained from the flow cytometry analysis, circulating monocytes were isolated from a group of enrolled SSc patients (11 SSc-ILD patients and 5 SSc patients without ILD) and HSs (n=5), differentiated *in vitro* to MDMs to evaluate the protein synthesis of TLR4 and M2 cell membrane markers.

**Figure 7:**
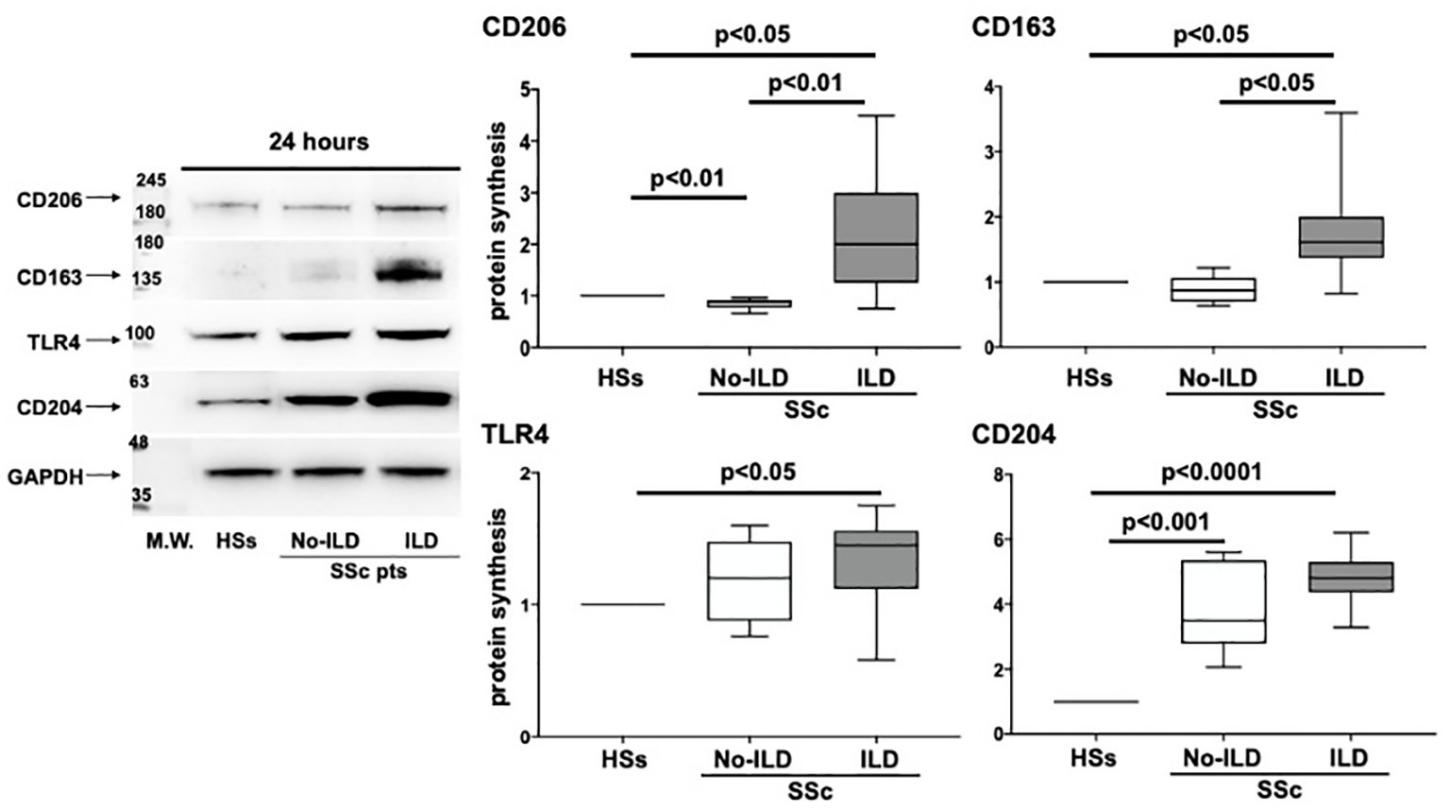
Evaluation by western blotting (WB) and related densitometric analysis of the protein synthesis of CD206, CD163, TLR4 and CD204 in cultures of monocyte-derived macrophages (MDMs) obtained from 5 voluntary healthy subjects (HS), 5 SSc patients without ILD (SSc no-ILD), and 11 SSc patients with ILD (SSc-ILD pts). The value of protein expression of CD206, CD163, TLR4 and CD204 was normalized to that of the corresponding glyceraldehyde 3-phosphate dehydrogenase (GAPDH) in cultured HS-MDMs, MDMs from SSc no-ILD, and MDMs from SSc-ILD. The resulting value of the protein expression of each molecule in cultured MDMs from SSc-ILD and from SSc no-ILD was compared with that obtained in cultured HS-MDMs (taken as unit value). The protein expression of each molecule obtained in cultured MDMs from SSc no-ILD and SSc-ILD represent the fold increase compared to the unit value of cultured HS-MDMs. Data are reported as median with a range of fold increase compared to HSs.

The Western blotting analysis showed that cultured MDMs obtained from SSc-ILD patients were characterized by a significant higher synthesis of CD206, CD163 and CD204 compared to MDMs of HSs (p<0.05; p<0.05; p<0.0001) (**Figure 7**). Moreover, cultured SSc-ILD MDMs showed a significantly higher synthesis of TLR4 compared to HSs (p<0.05) (**Figure 7**). Of note, the synthesis of CD206 and CD163 was significantly higher in cultured MDMs of SSc-ILD patients than in MDMs of SSc patients without ILD (p<0.01 and p<0.05, respectively) (**Figure 7**). No significant differences in the TLR4 and CD204 protein synthesis between SSc-ILD and SSc patients without ILD were observed (**Figure 7**).

## 4 Discussion

Current study highlighted a significant prevalence of alveolar macrophages characterized by M2 phenotype in SSc-ILD lung samples versus controls.

As matter of fact, the presence of cells expressing CD204, CD163 and CD206 in the same areas characterized by CD68 positivity, resembled what recently observed in the skin of SSc-patients (15).

However, M1/M2 paradigm of macrophage polarization further does not fully explain the complexity of this biological process in course of SSc (33). Indeed, for the first time, cells highly positive for TLR4 (M1 marker) and M2 markers were detected in the same areas (alveoli) of SSc-ILD samples, strongly suggesting the possible presence of hybrid TLR4^+^M2 macrophages (CD68^+^CD80^-^CD86^-^TLR4^+^CD163^+^CD204^+^CD206^+^cells) as a prevalent population at least in SSc-ILD.

Similarly, TLR4^+^M2 monocytes (CD1c^-^CD80^-^TLR4^+^CD163^+^CD204^+^CD206^+^cells) have been also identified in peripheral blood of SSc patients among the monocyte population (CD14^+^ cells).

It is also notable that the absence of CD1c, as marker of dendritic cells type II, excluded the presence of CD14^+^CD1c^+^ dendritic cells allowing a precise characterization of such peripheral monocytes (34, 35).

Of note, TLR4 is an innate immune activator that recognizes not only lipopolysaccharide, but also endogenous ligands of “damage-associated molecular patterns” (DAMPs) (36). One of the most frequent DAMPs in the course of SSc are extracellular matrix components, such as tenascin C and fibronectin-EDA (37). Once recognized by TLR4, these molecules activate a signaling pathway that includes the accessory protein MD2 and finally ends with the differentiation of tissue fibroblasts into active myofibroblasts, further supporting the fibrotic process (38).

SSc-ILD is currently the main cause of death of SSc-patients (39). The golden standard for SSc-ILD detection is HRCT, even though not worldwide available to screen each patient with SSc. NVC is a safe technique that allows the magnification of the diameter of skin capillaries up to 200-fold and identifies specific SSc abnormalities, classically divided into scleroderma patterns “early”, “active” and “late” (40). Scleroderma “late” NVC pattern is associated with more advanced lung involvement and the role of NVC for SSc-ILD monitoring is matter of growing research (41–43).

Beyond instrumental examinations, several biomarkers have been proposed to identify SSc-ILD, such as the measurement of serum concentrations of alveolar epithelial proteins (i.e., surfactant proteins A and D, Krebs von den Lungen-6 antigen), chemokines and cytokines (CCL2, CCL18, CXCL10), metalloproteinases, acute phase cytokines (i.e., IL-6), but their application in daily practice is still limited (44).

Therefore, the characterization of the phenotype of circulating monocytes and tissue macrophages in SSc-ILD patients has interesting potential to reflect organ involvement during the disease (23).

In current study, hybrid TLR4^+^M2 macrophages (CD68^+^CD80^-^CD86^-^TLR4^+^CD163^+^CD204^+^CD206^+^cells) were found in SSc-ILD samples of patients treated by different standard immunosuppressive therapies, including mycophenolate mofetil, calcineurin inhibitors (cyclosporine A and tacrolimus), basiliximab (anti-CD25) and alemtuzumab (anti-CD52), but none of these drugs are currently known to play an effect on M1/M2 macrophage polarization (45).

On the other hand, peripheral monocytes were collected by another cohort of SSc patients, again treated with different standard immunosuppressive therapies, such as mycophenolate mofetil, cyclophosphamide and rituximab, according to their disease condition and severity, again without significant differences between populations. Of note, the percentage of circulating hybrid TLR4^+^M2 and M2 monocytes was significantly higher in SSc patients with ILD than in those without ILD. Moreover, the fact that M2 macrophages expressing TLR4 are important in SSc-ILD was also confirmed in this study by the capability of cultured MDMs obtained from SSc-ILD patients to highly produce M2 phenotype markers CD206, CD163 on their cell membrane compared to cultured MDMs of SSc patients without ILD.

However, in the cohort of SSc patients enrolled for flow cytometry analysis, three of them (7.5% of total patients) were treated with nintedanib, an antifibrotic tyrosine kinase inhibitor licensed for the treatment of SSc-ILD and recently described as interfering with M2 polarization (46–48). Despite the limited number of these patients, their percentage of circulating M2 and hybrid TLR4^+^M2 monocytes was lower than other SSc-ILD patients not treated with nintedanib, suggesting the capability of the drug to reduce the percentage of these circulating monocytes, which might play a role in the fibrotic process of SSc-ILD.

Indeed, nintedanib downregulates the expression of M2 markers, including CD206, in primary cultures of human MDMs controlled by human recombinant colony-stimulating factor 1 (46). The antifibrotic effect of nintedanib was recently tested in cultured MDMs obtained from SSc-ILD patients; these cells were characterized by a profibrotic M2 phenotype with a significantly high expression of cell membrane (CD204, CD206 and CD163) and functional markers of profibrotic M2 phenotype (MerTK and TGFβ1) compared to cultured MDMs obtained from HSs and SSc patients without ILD (47).

Interestingly, the treatment with nintedanib significantly reduced both gene and protein expression of all these M2 markers (48). The effects of nintedanib on M2 macrophage polarization further support the overall antifibrotic effect, hindering the transition of fibrocytes into myofibroblasts (48).

New antifibrotic/immunosuppressive drugs are also being studied for their effect on macrophage polarization. Pirfenidone (not used in current study population) is another drug licensed for the treatment of idiopathic pulmonary fibrosis, currently under investigation for SSc-ILD. Pirfenidone attenuates fibroblast proliferation, production of proteins and cytokines associated to fibrosis and the increase in biosynthesis and accumulation of extracellular matrix in response to TGF-β (49). Of note, in mouse models, pirfenidone reduced M2 polarization, downregulating transcription factor NF-kB (50, 51).

Janus kinase inhibitors are immunosuppressive synthetic molecules that downregulate the activation of janus kinases (JAK1, JAK2, JAK3) and tyrosine kinase 2.

These drugs are not licensed for the treatment of SSc-ILD, but *in vitro* studies on monocyte-derived macrophages from healthy donors and on lung biopsies of hypochlorous acid mouse model of SSc-ILD demonstrated a significant effect in downregulating M2 polarization (52).

Although the presence of TLR4^+^M2 hybrid population of monocytes/macrophages in SSc-ILD patients seems to be clearly tested, the study has some limitations.

First, a single immunostaining was performed for each lung tissue section, without conferring absolute certainty that all the investigated markers are always expressed by the same cells; however, their overlap was evident and confirmed with several tests. In any case, a further analysis with multiplex immunoassays and confocal microscopy is mandatory and already planned to confirm the presence of cells co-expressing M1/M2 markers in SSc-ILD samples.

In addition, the direct lineage of these cells should be clarified, investigating if they derive from lung resident macrophages or peripheral blood monocytes or both (i.e., transcriptomic analysis) (53). Similarly, the cytokine production from TLR4^+^M2 macrophages will be also analyzed and characterized.

Furthermore, the study involved two cohorts of SSc-ILD patients. The lung samples were obtained from patients with very advanced SSc-ILD before lung transplantation. On the other hand, due to the mentioned technical issues, blood samples were obtained from a second cohort of SSc-ILD patients, with presence of both altered functional pulmonary tests and major presence of advanced “late” NVC pattern, that associate with advanced fibrotic lung involvement (41–43).

Therefore, a further lung sampling in SSc-ILD patients with less severe/progressed lung involvement, should be an additional step to evaluate possible differences in the presence of TLR4^+^M2 macrophages in early versus advanced disease.

As matter of fact, further studies are ongoing to search for a possible relationship between the observed macrophages phenotypes clustering and the interaction with fibrocytes, including also a role for specific immunosuppressive concomitant treatments (54).

In conclusion, TLR4^+^M2 positive monocytes (CD1c^-^CD80^-^TLR4^+^CD163^+^CD204^+^CD206^+^cells) and TLR4^+^M2 positive macrophages (CD68^+^CD80^-^CD86^-^TLR4^+^CD163^+^CD204^+^CD206^+^cells) represent a hybrid cell cluster that seems to be expressed on both peripheral blood, and for the first time, in the cellular infiltrate of lung samples from SSc-ILD patients. Therefore, the detection of TLR4^+^M2 monocytes in blood samples, might represent a further potential new biomarker of such clinical complication and generally of progressive fibrosis (other immune mediated diseases complicated by ILD are under evaluation).

The correlation between the percentage of circulating TLR4^+^M2 monocytes and the severity of the fibrotic tissue and organ involvement in SSc patients is currently being evaluated.

## Data Availability

The raw data supporting the conclusions of this article will be made available by the authors, without undue reservation.
