# Supplementary material for: Prevalence of hybrid TLR4+M2 monocytes/macrophages in peripheral blood and lung of systemic sclerosis patients with interstitial lung disease
Source: Front Immunol. 2024 Nov 20;15:1488867. doi: 10.3389/fimmu.2024.1488867 (PMC11615060; doi:10.3389/fimmu.2024.1488867)
Supplement: Supplementary file 1 [file DataSheet1.docx]

**Supplementary Material 1**

**Primary antibodies for histopathological assessment**

Following primary antibodies were used for single immunostaining:

- Mouse anti-human CD68 (pan specific macrophage marker, Clone KP1, Biocare, Pacheco, California, USA) – dilution 1:50
- Mouse anti-human CD80, CD86, TLR4 (M1 markers - Santa Cruz Biotechnology, Dallas, Texas, USA) – dilution 1:50
- Rabbit anti-human CD163 and CD206 (M2 markers - Cell Signaling Technology, Danvers, Massachusetts, USA) – dilution 1:50
- Mouse anti-human CD204 (M2 marker) (cod. sc-166184, Santa Cruz Biotechnology, Dallas, Texas, USA) – dilution 1:50.
- 3’3’-diaminobenzidine (DAB) enhanced liquid substrate system tetrahydro-chloride (cod. D3939; Sigma-Aldrich, Burlington, Massachusetts, USA).

**Primary antibodies for flow cytometry**

For each enrolled systemic sclerosis patient and healthy subject, a volume of 3mL of peripheral whole blood was collected in a lithium-heparin vacutainer and processed until 1 hour. For flow cytometry analysis, a volume of 0.1mL of peripheral whole blood was transferred into a polystyrene tube and the conjugated primary antibodies were added as follows:

- Mouse anti-human conjugated antibody CD45-Krome Orange (clone J33; leukocyte marker, Beckman Coulter, Brea, California, USA) – dilution 1:10.
- Mouse anti-human conjugated antibody CD16-APC (clone 3G8; monocyte marker, Beckman Coulter, Brea, California, USA) – dilution 1:10.
- Mouse anti-human conjugated antibody CD14-FITC (REAffinity, clone REA599; monocyte marker, Bergisch Gladbach, Germany) – dilution 1:50.
- Mouse anti-human conjugated antibody CD204-PE (REAffinity, clone REA460; M2 macrophage marker, Bergisch Gladbach, Germany) – dilution 1:50.
- Mouse anti-human conjugated antibody CD206-PerCPVio700 (clone DCN228; M2 macrophage marker, Bergisch Gladbach, Germany) – dilution 1:10.
- Mouse anti-human conjugated antibody CD163-PEVio615 (REAffinity, clone REA 812; M2 macrophage marker, Bergisch Gladbach, Germany) – dilution 1:50.
- Mouse anti-human conjugated antibody CD80-PEVio770 (clone 2D10; M1 macrophage marker, Bergisch Gladbach, Germany) – dilution 1:10.
- Mouse anti-human conjugated antibody TLR4-BV421 (clone TF901; M1 macrophage marker, Becton Dickinson-BD Biosciences, Franklin Lakes, New Jersey, USA) – dilution 1:20.
- Mouse anti-human conjugated antibody CD1c-APC Cy7 (clone L161; dendritic cell marker, Biolegend) – dilution 1:20.
